# SUV-quantification of physiological lung tissue in an integrated PET/MR-system: Impact of lung density and bone tissue

**DOI:** 10.1371/journal.pone.0177856

**Published:** 2017-05-31

**Authors:** Ferdinand Seith, Holger Schmidt, Sergios Gatidis, Ilja Bezrukov, Christina Schraml, Christina Pfannenberg, Christian la Fougère, Konstantin Nikolaou, Nina Schwenzer

**Affiliations:** 1 Diagnostic and Interventional Radiology, Department of Radiology, Eberhard Karls University, Hoppe-Seyler-Straße 3, Tuebingen, Germany; 2 Preclinical Imaging and Radiopharmacy, Laboratory for Preclinical Imaging and Imaging Technology of the Werner Siemens Foundation, Department of Radiology, Eberhard Karls University, Hoppe-Seyler-Straße 3, Tuebingen, Germany; 3 Max Planck Institute for Intelligent Systems, Spemannstr. 38, Tuebingen, Germany; 4 Nuclear Medicine, Department of Radiology, Eberhard Karls University, Hoppe-Seyler-Straße 3, Tuebingen, Germany; Wayne State University, UNITED STATES

## Abstract

**Purpose:**

The aim of the study was to investigate the influence of lung density changes as well as bone proximity on the attenuation correction of lung standardized uptake values (SUVs).

**Methods and materials:**

15 patients with mostly oncologic diseases were examined in 18F-FDG-PET/CT and subsequently in a fully integrated PET/MR scanner. From each PET dataset acquired in PET/MR, four different PET reconstructions were computed using different attenuation maps (μ-maps): i) CT-based μ-map (gold standard); ii) CT-based μ-map in which the linear attenuation coefficients (LAC) of the lung tissue was replaced by the lung LAC from the MR-based segmentation method; iii) based on reconstruction ii), the LAC of bone structures was additionally replaced with the LAC from the MR-based segmentation method; iv) the vendor-provided MR-based μ-map (segmentation-based method). Those steps were performed using MATLAB. CT Hounsfield units (HU) and SUVmean was acquired in different levels and regions of the lung. Relative differences between the differently corrected PETs were computed.

**Results:**

Compared to the gold standard, reconstruction ii), iii) and iv) led to a relative underestimation of SUV in the posterior regions of -9.0%, -13.4% and -14.0%, respectively. Anterior and middle regions were less affected with an overestimation of about 6–8% in reconstructions ii)–iv).

**Conclusion:**

It could be shown that both, differences in lung density and the vicinity of bone tissue in the μ-map may have an influence on SUV, mostly affecting the posterior lung regions.

## Introduction

In positron emission tomography (PET), the correction of the attenuation (attenuation correction, AC) of annihilated photons on their way through the patient's body plays a key role in quantifying the local tracer-uptake, expressed as standardized uptake value (SUV) [1]. In positron emission tomography/computed tomography (PET/CT), a direct transformation of Hounsfield Units (HU) into the attenuation of 511keV-photons is possible, leading to precise linear attenuation coefficients (LACs) of the tissue which is used to compute an attenuation map (μ-map). Since the signal intensity in magnetic resonance (MR) imaging cannot be directly related to photon attenuation, an analogous procedure is not possible in integrated PET/MR-systems. To date, several approaches have been proposed to overcome this drawback. The most commonly used one is the segmentation-method [2] as it is used e.g. in the Siemens Biograph mMR. In principle, it separates the body into 3–4 different tissue types based on a T1-weighted MR sequence (air, lung, soft tissue and fat) and corresponding LACs are assigned. As the segmentation-method is based on standard MR sequences, bone is routinely not recognized due to low signal intensity of osseous tissue and therefore not considered in the μ-map. However, bone causes the highest attenuation of photons in the human body which can lead to significant underestimation of SUV in regions with a high amount of bone tissue [3]. Another limitation lies in the fact that the segmentation-method relies on the attribution of four predefined tissue classes to all body parts, however, only one single LAC is attributed to the whole lung. This LAC for lung is patient-independent and does not account for regional lung density changes. Thus, both inter-subject as well as regional differences in lung tissue may not be considered for AC using the segmentation-method in the Biograph mMR.

PET/MRI can be used as an alternative to PET/CT in staging oncologic patients [4], providing lower radiation exposure for patients and functional imaging techniques such as diffusion weighted imaging. It is known that—compared to CT—MR is inferior in detecting small lung nodules [5] and therefore, the PET information in PET/MR might even be more important for the detection and characterization of pulmonary nodules than it is in PET/CT [6]. Previously conducted studies could show that the segmentation-method might not be sufficient for reliable LAC of lung tissue [7–15]. Keereman et al. [9, 10] showed in simulations, that misclassifications in lung and cortical bone tissue can have a significant impact on PET quantification. However, the potential clinical impact of these effects has not yet been evaluated in systematic studies.

Therefore, the aim of this study was to assess the accuracy of the regional SUV quantification based on the segmentation-method compared to the CT-based attenuation correction, with focus on the impact of regional lung density changes as well as the anatomical vicinity of bone.

## Material and methods

### Patient cohort

In a prospective study conducted between March 2011 and October 2013, 200 patients underwent a clinically indicated PET/CT and subsequent PET/MR examination in a fully integrated PET/MR system. The study was designed as a feasibility study to compare PET/MR with PET/CT including dedicated examinations of the brain, the upper and lower abdomen as well as whole body examinations. The study was approved by the German Federal Institute for Drugs and Medical Devices as well as the local ethics committee. Written informed consent was obtained from all patients concerning both examination and the scientific evaluation of their data. The first 60 patients had to be excluded due to a vendor-provided software update that could influence PET quantification. Furthermore, 19 examinations had to be excluded due to technical problems such as strong artifacts or registration problems. From the remaining 121 cases, 21 PET/MR examinations had a diagnostic lung imaging protocol from whom 15 were investigated with 18F-FDG and were thus included for evaluation (8 female, mean age 56.7 ± 13.7 years). Lung regions with consolidations or malignant lesions (e.g. infiltrates, dystelectasis, atelectasis, metastases, pleural carcinomatosis) were excluded. Most of the patients had known oncologic diseases: oesophageal cancer (n = 2), head-and-neck-cancer (n = 1), melanoma (n = 1), lymphoma (n = 1), ovarian cancer (n = 2), pulmonary cancer (n = 2), breast cancer (n = 2), thyroid cancer (n = 1). The other participants were suffering from fever of unknown origin (n = 2) and vasculitis (n = 1).

In order to identify patients with severe lung emphysema as a potential influencing factor on the SUV measurement, an additional CT-based analysis of lung density was performed in all included patients using the imaging software Pulmo3D (syngo.via, Siemens Healthcare GmbH, Erlangen, Germany). Each lung was separated into three segments (upper part, middle part and lower part) and the mean lung density (MLD) was measured in each segment. MLD below -950 HU was considered as pathologic and suspicious of severe emphysema [16, 17].

### Hybrid examinations

All PET/CT scans were performed on an integrated scanner with 64-slice CT technology (Biograph mCT, Siemens Healthcare GmbH, Erlangen, Germany). Depending on the clinical indication, a diagnostic contrast-enhanced CT (contrast media: Ultravist 370, Bayer Healthcare, n = 8) or a low-dose unenhanced CT (n = 7) was performed. While the whole-body CT examination was performed in end-expiratory breath-hold in order to acquire a μ-map, an additional CT scan of the lung in inspiratory breath-hold was performed (120 kv, 2 mm axial reconstruction slice thickness, convolution kernel B31f or B30f). Before intravenous injection of ^18^F-FDG, patients fasted for at least 6 h. Tracer injection took place before the PET/CT examination. Average injected dose of 18F-FDG was 351.8 ± 23.7 MBq, average uptake time for PET/MRI was 130 ± 15.6 min. The injected activity was sufficient for subsequent PET/MR imaging which was performed on average 68.7 ± 13.7 minutes after the PET/CT examination had started. Thus, no additional tracer injection had to be performed. All combined PET/MR examinations were performed on a fully integrated whole-body PET/MR system (Biograph mMR, Siemens Healthcare GmbH, Erlangen, Germany) which is able to acquire PET and MR simultaneously. To create a segmentation-based μ-map, a 3D T1-weighted spoiled gradient-echo sequence (VIBE) was performed in end-expiratory breath-hold, with Dixon-based fat-water separation and with the following sequence parameters: time of acquisition (TA), 19 s; matrix size, 79 x 192; repetition time (TR), 3.6 ms; excitation angle, 10°; echo times (TE), TE1 = 1.23 ms, TE2 = 2.46 ms; pixel size, 2.6 x 2.6 x 2.6mm; bandwidth, 965Hz/pixel; 128 slices per slab; parallel imaging acceleration factor 2. The μ-map is calculated by the system using the compartments air (0cm^-1^), lung (0.0224cm^-1^), fat (0.0854cm^-1^) and soft tissue (0.1cm^-1^). In all PET/MR exams, a coronal short time inversion recovery (STIR) sequence was acquired in free breathing during PET acquisition with TR/TE, 3000 ms/81 ms; inversion time (TI), 220 ms; excitation angle, 120°; bandwidth, 383 Hz/pixel; matrix size, 145 x 256; resolution, 2.0 x 1.8 x 5.0mm; parallel imaging acceleration factor 3; averages 2; TA, 2 min 13 s. Patients’ arms were positioned above the head in PET/CT and alongside the abdomen in PET/MR.

Other sequences were chosen depending on the clinical question. Based on AC templates, the AC for the spine array coil and the patient table was performed automatically. For MR signal detection, a standard phased-array body coil was used, optimized for minimal photon attenuation. This flexible coil was not considered in the μ-maps.

### Reconstruction of PET images from PET/MR using different attenuation correction modes

The CT-based μ-map was derived from the CT dataset of the PET/CT examination, the CT table was removed manually using MATLAB. The CT image was non-rigidly registered to the T1-weighted in-phase MR images which are also used to calculate the MR-based attenuation μ-map and resampled to the same spatial resolution. HU values were converted to LAC values using a piecewise bilinear transformation also used in the PET/CT scanners [18]. This was performed with the software tool elastix [19]. Since the CT and MR were acquired with different arm positions, the arm regions in the CT-derived attenuation maps were replaced using the original attenuation map created by the system [20].

**Fig 1:**
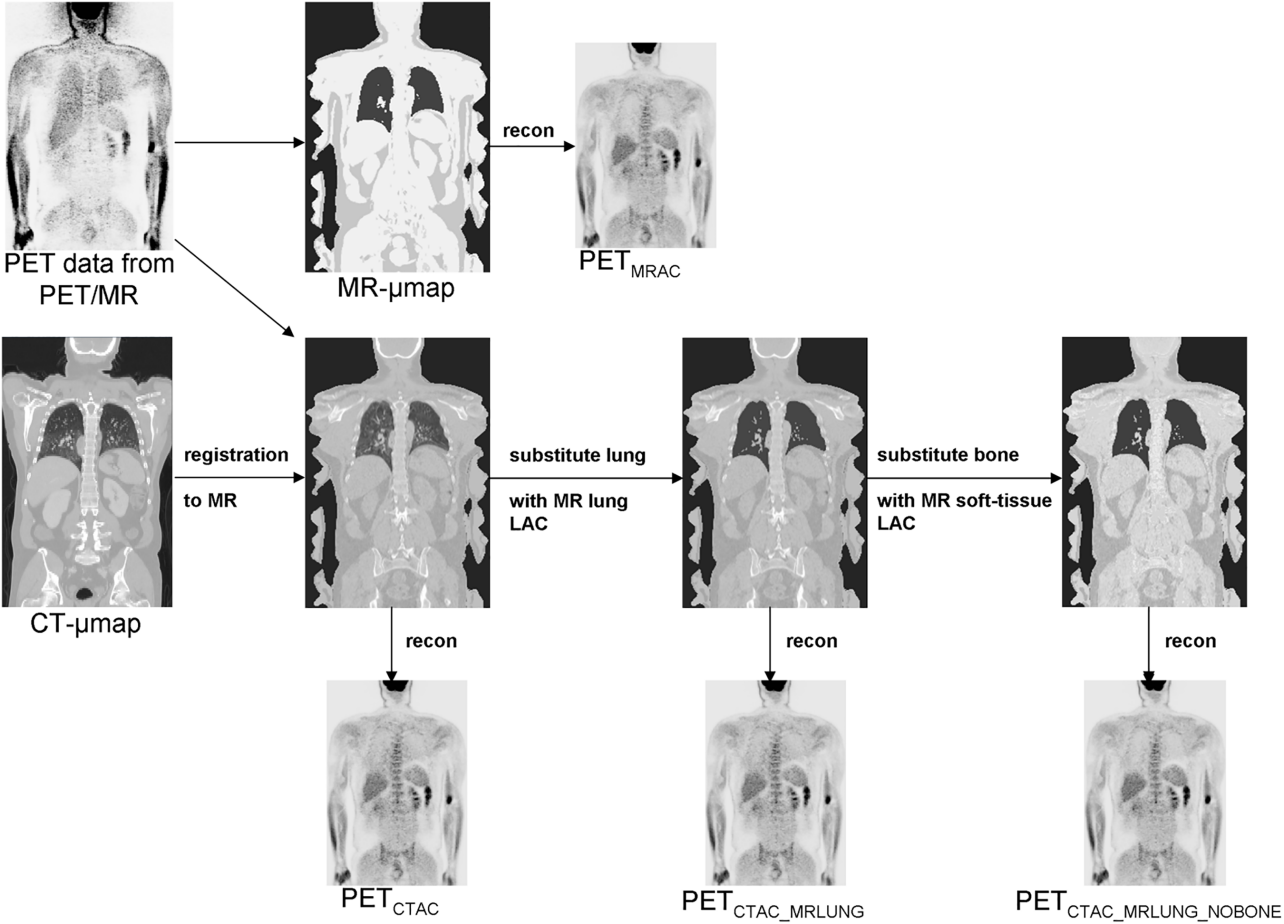
Overview of the workflow of reconstruction of PET images using different attenuation correction modes.

From each PET dataset acquired in PET/MR, four different PET reconstructions were computed using different μ-maps: i) a CT-based μ-map (PET_CTAC_, gold standard); ii) a CT-based μ-map in which the LAC of the lung tissue was replaced by the lung LAC from the MR-based segmentation method (PET_CTAC_MRLUNG_); iii) based on reconstruction ii), the LAC of bone structures was additionally replaced by the LAC of soft tissue from the MR-based segmentation method (PET_CTAC_MRLUNG_NOBONE_); iv) the vendor-provided MR-based μ-map (PET_MRAC_, segmentation-based method). The detailed workflow is depicted in Fig 1.

To correct for different arm positions in PET/CT (elevated arms) and PET/MR, arms were removed in the CT datasets and replaced with arm regions of the corresponding MR-based μ-map. These steps were performed using MATLAB (The MathWorks Inc., Natick, MA, USA), results were controlled visually.

Masks of the lung volumes from these CT-based μ-maps were created using a region growing algorithm implemented in Imagine (Version 2.0; http://www.mathworks.com/matlabcentral/fileexchange/40440-imagine). The LACs of all voxels within this mask were set to the lung LAC of the MR-based μ-map (0.022 cm^-1^) using MATLAB. Finally, voxels of bony structures (LAC > 0.10 cm^-1^) were set to soft-tissue LAC of the MR-based μ-map (0.1 cm^-1^) using MATLAB.

**Fig 2:**
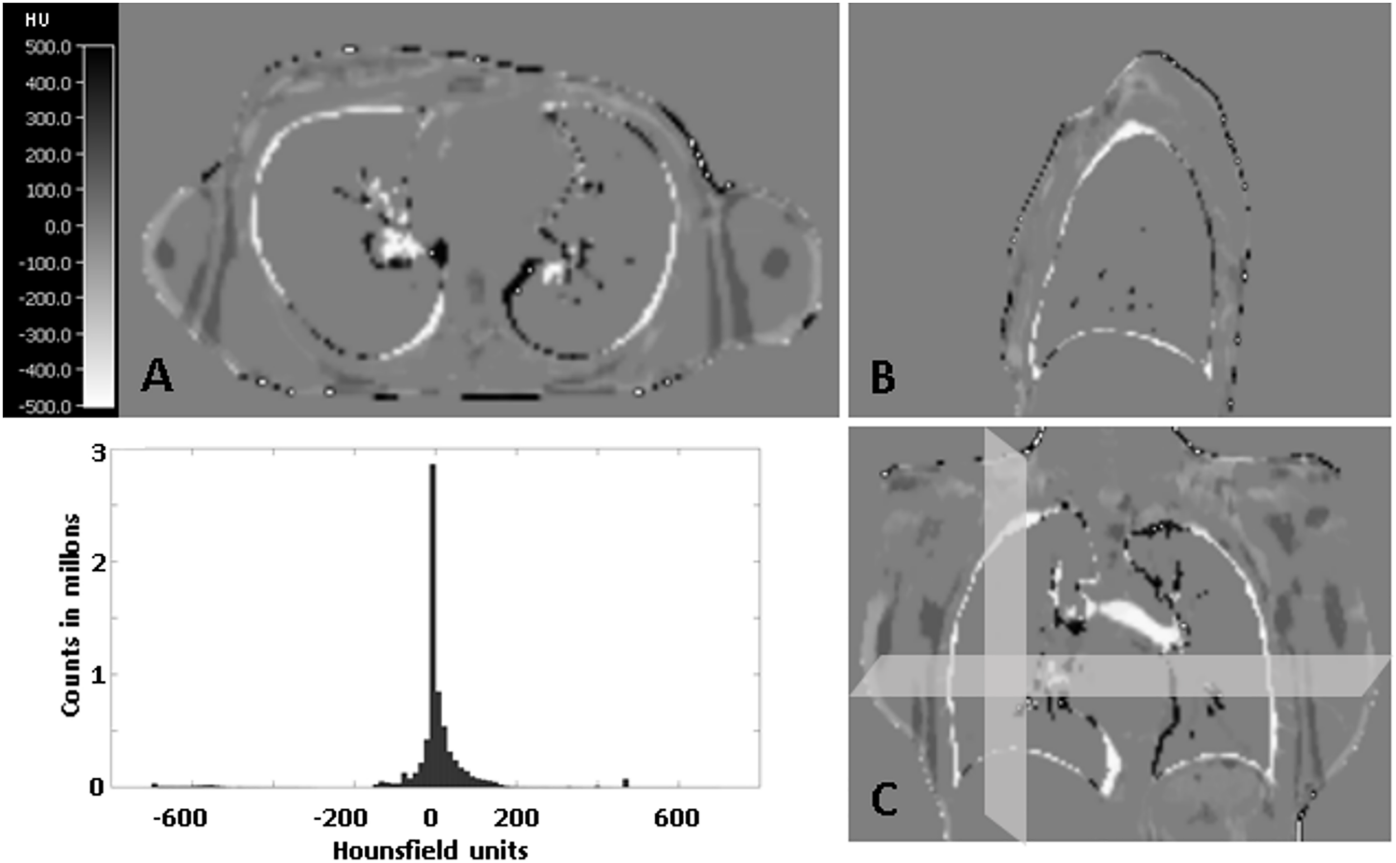
Example of a subtraction image of the MR-based μ-map and the registered CT-based μ-map of a patient in which the LACs from the lung and the bone tissue were replaced by the LACs from the MR- μ-map in axial view (A), sagittal view (B) and coronal view (C). The histogram analysis of all patients shows that most of the voxels within the patient from the subtraction images provide HU of around 0 (>90% of the voxels show a deviation of more than 10% from the maximum value (0.1cm^-1^)) which stands for the high precision of the performed registration.

To assess the precision of the registration of CT images to MR images, we performed a voxelwise subtraction of the CT-based μ-maps in which the LACs of lung and bone were replaced (μ-maps iii)) from the MR-based μ-map. A histogram analysis of the voxels within each patient was performed after segmentation of the patient from the background. Results are given in Fig 2.

PET data were reconstructed with the different μ-maps using the PET reconstruction software provided by the vendor (e7-tools, Siemens Healthcare GmbH, Knoxville, TN, USA). A 3D ordered-subset expectation-maximization algorithm with 2 iterations, 21 subsets, matrix size 256 x 256, Gaussian filtering of 4 mm was used.

### Image analysis

**Fig 3:**
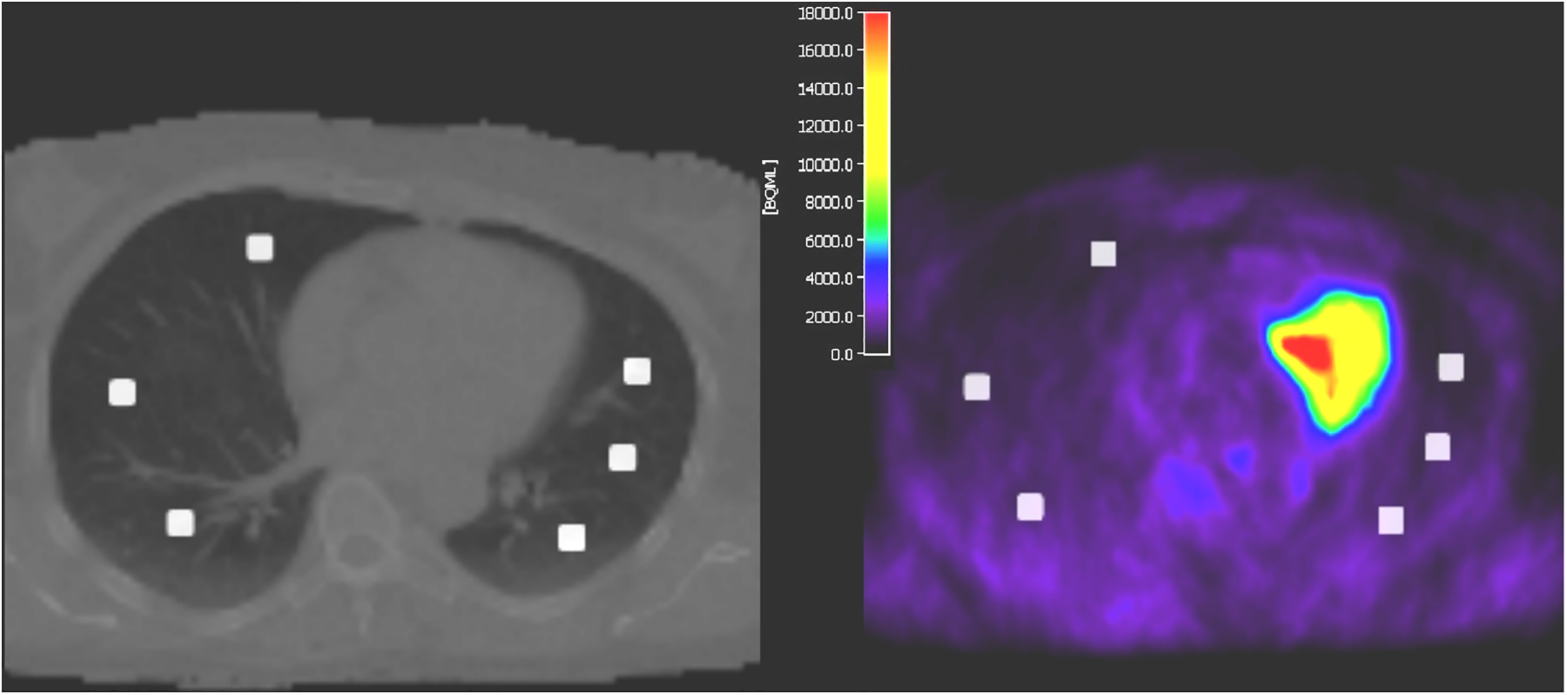
Example of ROI placements in the basal lung parts. Left hand side: ROIs superposed with coregistered μ-map from CT. Right hand side: ROIs superposed with the correlating PET.

Image analysis was performed using the imaging software PMOD (PMOD Technologies Ltd., Zurich, Switzerland). The STIR sequences as well as the MR and CT μ-maps from each patient were rigidly registered to the corresponding PET data from the PET/MR system. 12 ROIs with a diameter of 1 cm were placed in physiological lung tissue of each patient. For each lung, three were set at the level of the hilum (anterior, middle, posterior) and three were set in the basal lung (anterior, middle, posterior). Fig 3 shows an example for the ROI placement.

μ-maps from MR as well as the computed μ-maps from CT were rigidly overlaid to the PET to avoid positioning of ROIs in artifacts. The ROIs exported from PMOD were copied to the different corresponding PETs and to the CTs from PET/CT. The relative difference of SUVmean in percent in the different lung parts of the different PET images X to the reference PET_CTAC_ was defined as: (SUVmean (X)—SUVmean (PET_CTAC_)) / SUVmean (PET_CTAC_) * 100. Moreover, Hounsfield units (HU) were measured in each ROI in the CT. Since CT contrast agents can have an influence on CT HU in the lung [21, 22], we also provided a separate analysis of HU differences in lung regions for unenhanced and enhanced CT data.

CT HUs and the SUVmean between the differently reconstructed PETs and the relative differences of HUs and SUVmean between the different regions were statistically compared using analysis of variance with Bonferroni post-hoc correction. P-values < 0.05 were considered significant (SPSS Statistics 19, IBM, Armonk, NY).

## Results

In the MLD evaluation, lung density was higher than -950 HU in all evaluated segments so that per definition, no emphysematous lung areas were present in the evaluated patient group. HU values are given in Table 1.

**Table 1:** Overview of mean values and SD of measured Hounsfield units (HU) of CTs from PET/CT in different lung regions. A gradient from anterior to posterior can be seen in both contrast-enhanced and non-contrast-enhanced CTs, pronounced between the middle and posterior regions. Values of contrast-enhanced CTs and non-contrast-enhanced CTs as well as the differences between the anterior and posterior regions did not differ significantly.

**Hounsfield Units all patients (n = 15)**			
	Anterior	Middle	Posterior
Mean±SD	-779±64	-746±1	-685±118
**Hounsfield Units contrast-enhanced CT (n = 8)**			
Mean±SD	-770±80	-732±86	679±128
Mean difference anterior-posterior: 94 HU			
**Hounsfield Units non-contrast-enhanced CT (n = 7)**			
Mean±SD	-788±40	-761±87	-691±108
Mean Difference anterior-posterior: 97 HU			

HUs of contrast-enhanced and non-contrast-enhanced CTs and the differences between the anterior and posterior regions did not differ significantly between these two groups (p = 0.17 and p = 0.92, respcectively). A gradient in CT HUs from the anterior to the posterior region is visible with higher HUs in the posterior regions. The SUVmean values are given in Table 2.

**Table 2:** SUVmean including standard deviations in different regions and different PET images. P-values describe the significance of differences between the anterior and posterior lung regions (p-value a.-p.) in the respective PET.

	Anterior	Middle	Posterior	p-value a.-p.
PET_CTAC_	0.31 ± 0.1	0.33 ± 0.1	0.47 ± 0.2	4.7*10^−9^
PET_CTAC_MRLUNG_	0.32 ± 0.1	0.33 ± 0.1	0.42 ± 0.1	3.8*10^−6^
PET_CTAC_MRLUNG_NOBONE_	0.33 ± 0.1	0.32 ± 0.1	0.39 ± 0.1	6.2*10^−6^
PET_MRAC_	0.32 ± 0.1	0.32 ± 0.1	0.39 ± 0.1	2.4*10^−4^

**Fig 4:**
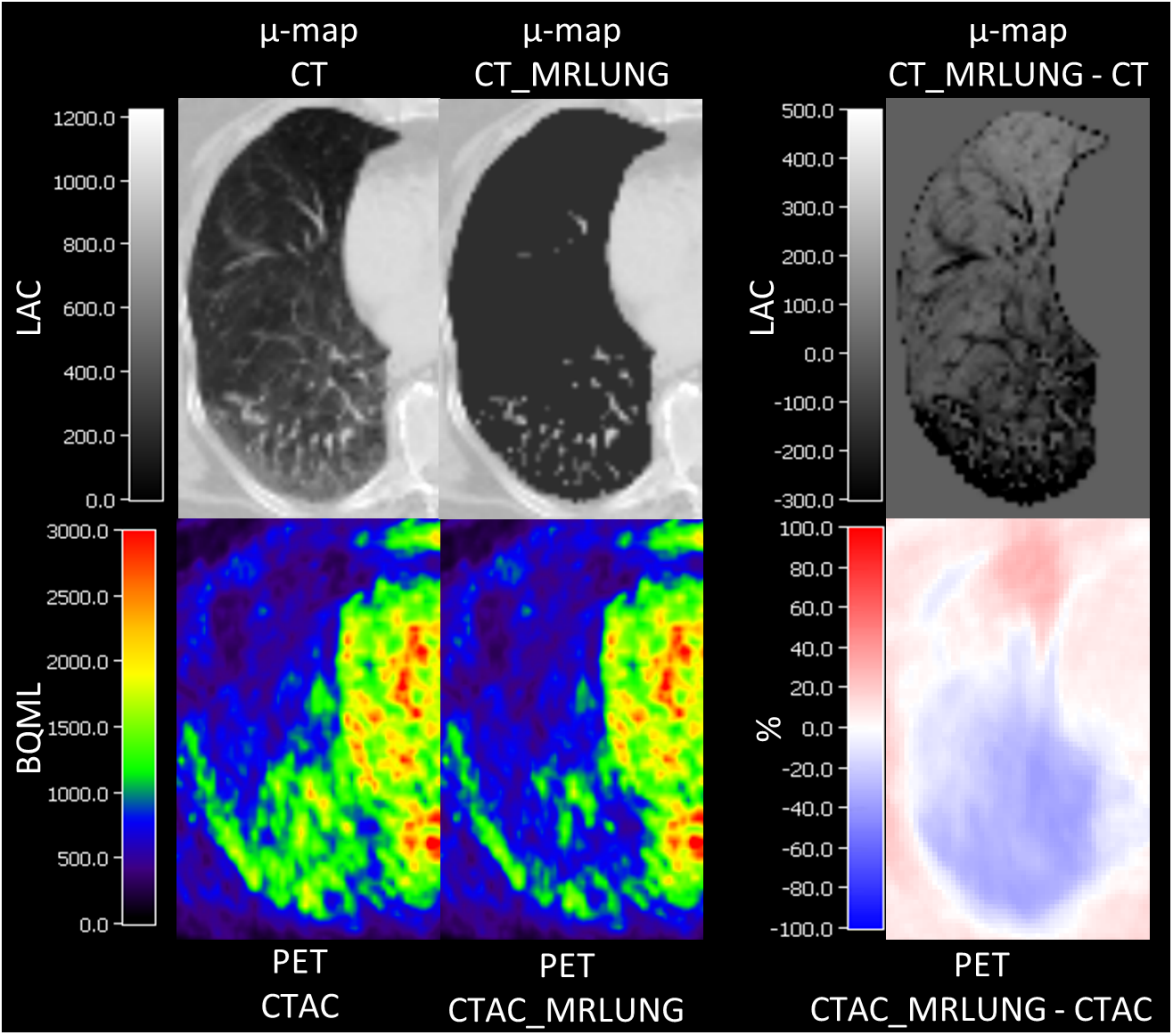
Typical example of an axial view of the right lung of a patient to demonstrate the effect of the replacement of the CT-based LACs of lung tissue (μ-map CT) with the LAC from the MR-based segmentation method from the Biograph mMR (μ-map CT_MRLUNG). To show the differences of LACs, a subtraction map is given in the upper row on the right-hand side (μ-map CT_MRLUNG—CT); as one can see, the subtraction of μ-map CT from μ-map CT_MRLUNG leads to an underestimation of LACs in the posterior regions and an overestimation in the anterior regions (given in LACs). This results in higher SUVs in PET_CTAC as compared to PET_CTAC_MRLUNG in the posterior regions, or, in other words, an underestimation of SUVs in the posterior regions of PET_CTAC_MRLUNG as shown in the subtraction image PET CTAC_MRLUNG–CTAC.

A gradient of SUV from anterior to posterior is clearly visible with decreasing intensity from PET_CTAC_ to PET_MRAC._ The relative differences between the differently corrected PETs are shown in Table 3. An example is given in Fig 4.

**Table 3:** Overview of relative differences in % between the differently reconstructed PETs and regions. Note the differences of SUV-underestimation in the posterior lung parts while the overestimations in the anterior regions do not change significantly. P-Values describe the significance of differences between the particular lung regions in the different PET images. The SUVs of PET_CTAC_MRLUNG_NOBONE_ and PET_MRAC_ images did not differ significantly in any region.

	Anterior	Middle	Posterior
**(PET**_**CTAC_MRLUNG**_**-PET**_**CTAC**_**)/PET**_CTAC_*100			
Mean ± SD	6.2±12.3	0.7±12.5	**-9.0±14.3**
p-value	0.29	0.81	0.05
**(PET**_**CTAC_MRLUNG_NOBONE**_**-PET**_**CTAC**_**)/PET**_CTAC_*100			
Mean ± SD	8.0±13.9	0.2±13.7	**13.4±17.12**
p-value	0.15	0.63	0.003
**(PET**_**MRAC**_**-PET**_**CTAC**_**)/PET**_CTAC_*100			
Mean ± SD	5.7±13.0	-2.8±12.0	**-14.0±15.8**
p-value	0.33	0.33	0.003

The maximum relative percentage of overestimation of PET_MRAC_ compared to PET_CTAC_ was +35.4% (anterior part of the lung); the maximum relative percentage of underestimation of PET_MRAC_ to PET_CTAC_ was -48.9% (posterior part of the lung). In all reconstructed PETs, there was no significant difference between the measured SUVmean of the right and left lung.

The SUVmean for the different lung regions of the individual patients are given in S1–S4 Tables for PET_CTAC, PET_MRLUNG, PET_MRLUNG_NOBONE, PET_MRAC, respectively. The analysis of the lung densities and the respective Hounsfield units of the individual patients are given in S5 and S6 Tables, respectively.

## Discussion

It is well known that the density of lung tissue differs between individuals. Previously conducted studies could show that the interpatient variability and the differences between the right and left lung of a patient can influence the quantification in PET [9, 10, 15]. However, there are additional effects on lung tissue density, such as gravitational dependency, breathing position and underlying pathology in each patient [23–27].

To create a μ-map for PET/CT, the CT part is usually acquired in end-expiratory breath-hold and in supine position, which seems to provide best results in AC [28]. This offers different HU values, LAC and therefore a gradient of SUVmean for anterior, middle and posterior parts of the lung (Table 1 and Fig 4). In contrast, the segmentation-method for MR-based AC defines one single lung LAC for the entire lung and does not account for potential variances in patients’ individual lung density. This would imply that the lung tissue was very homogeneous. With the replacement of LACs of the CT-derived lung tissue with LAC, as provided in the segmentation–method (PET_CTAC_MRLUNG_), an overestimation of SUV in the anterior parts and a SUV underestimation in the posterior parts of the lungs in the resulting PET occurs, most probably because the gravitational dependency in the posterior parts of the lung is ignored (Fig 4). By an additional replacement of the LACs of bony structures with LAC of soft tissue of the MR-based μ-map (PET_CTAC_MRLUNG_NOBONE_), a further increase of SUV-underestimation compared to PET_CTAC_ in the posterior regions was observed. This suggests that the underestimation of SUV as it is seen in the posterior lung parts in the original PET_MRAC_ is not only caused by a gradient of lung tissue density from anterior to posterior, but also from the surrounding bone tissue, e.g., the spine and the costovertebral joints. In the anterior parts of the lung, the replacement of LACs of bony structures in the CT μ-map did not to have additional significant influence on the SUV in our evaluation, probably caused by the lower amount of surrounding bony tissue in those regions. There was no significant difference observed between SUVs of the PET_MRAC_ and PET_CTAC_MRLUNG_NOBONE_ images. This let suggest that differences in lung density and surrounding bone tissue are the most influencing factors on the SUV in the lung. Corresponding to the observations acquired in the relative differences, the gradient of SUVmean from anterior to posterior which can be seen in PET_CTAC_ is continuously decreasing with the replacement of lung-LAC and bone-LAC to PET_MRAC_. However, there is still a small gradient of tracer uptake from anterior to posterior seen in the PET_MRAC_. As the MRAC does not take differences of lung density into account this finding represents a slightly higher accumulation of ^18^F-FDG-PET tracer in the posterior regions. As we focussed our work on the influence of different attenuation maps on the quantification of tracer uptake in the lung, the evaluation of regional distribution of different PET tracers in physiological lung tissue was beyond the scope of our work.

Several studies demonstrated that PET/MR is reliable in staging lung cancer with no significant differences compared to PET/CT regarding the clinical impact [29–32]. However, modern oncology will ask for quantifiable and reproducible results of imaging technologies in the initial assessment of the disease, therapy monitoring and for evaluation of tumor response to treatment. As a consequence, PET information does not only serve for lesion detection but also for quantifying tracer uptake compared to reference points (e.g., the liver) as it is common, e.g., in lymphoma [33] but also in the recently proposed PERCIST criteria [34]. Atlas-based methods are expected to be implemented into vendor software for AC in fully integrated PET/MR-systems [20, 35]. This will lead to an implementation of bone tissue into the μ-map for attenuation correction of PET in PET/MR. Based on our results it can reasonably be assumed that this will lead to a reduction of SUV underestimation in the posterior lung regions which is partially caused by bone tissue. However, the deviation of SUV based on differences in lung density compared to PET/CT might still persist [14]. A gradient-adjusted μ-map for the lung as well as the correction of patient-specific differences [9, 10] might further reduce the system-based error of PET-quantification in the lung in fully integrated PET/MR-systems. Patient-based, individual differences might be corrected by measuring the lung density as proposed by Marshall et al. [11] or specific MR sequences like ultra-short echo time MR imaging [7, 12]. Another possibility to improve PET quantification in lung tissue has recently been proposed by Mehranian et al. by using continuous and patient-specific lung LACs, derived from time-of-flight (TOF)-PET [13].

Our study has several limitations: As the scope of our work was to investigate the influence of different methods of AC on the SUVmean in physiological lung tissue, our patient cohort did not contain a substantial number of patients with PET-avid lesions in the lung. A specific investigation of lung lesions and a potential clinical impact (e.g. lesion detection and PET quantification of lesions) will be the scope of future studies. However, it should be noticed that it is generally difficult to differentiate between respiratory motion effects, non-recognition of pulmonary lesions in the μ-map leading itself to SUV underestimation, misalignment between PET and the μ-map and the erroneous recognition of lung tissue and bone as it was elaborated in the present study. Moreover, one has to consider that PET/CT as gold standard may also be hampered in the evaluation of small lung lesions by these effects, which limits the significance of comparing PET/CT and PET/MR regarding lesion assessment. We only included lung regions which not obviously affected by diseases (in CT, MRI or PET) and, moreover, performed a measurement of lung density in CT in the different regions to make sure that we did not analyze patients with e.g. emphysema. However, this approach does not exclude all kinds of diseases which might influence the distribution of PET tracers. A more profound evaluation of lung tissue would include histological analysis or an investigation of healthy volunteers in PET which is ethically not feasible.

The patients`arms in PET_CTAC_ were replaced by the corresponding MR-segmented arms, which were not containing bone. This leads to a potential slight misinterpretation of PET in the shoulder girdle and the apex of the lungs which currently cannot be avoided. However, since all PET images of a patient were reconstructed with the same arms, this should not have influence on the relative differences. We used flexible MR coils for the examinations which are not attenuation corrected and which might therefore also have an impact on the anterior-posterior gradient found [36–38].

### Conclusions

In conclusion, it could be shown that both surrounding bone tissue and differences in lung density in the μ-map may have noticeable influence on SUV in physiological lung tissue, mostly affecting the posterior lung regions. This should be taken into account for PET/MR lung reading and for the comparison of PET/CT and PET/MR examinations.

## Supporting information

S1 Table SUVmean in different lung regions in PET_CTAC. This table shows the SUVmean from ROI analysis in the different lung regions in the PET image reconstructed with the attenuation map from PET/CT (PET_CTAC).
(PDF)

S2 Table SUVmean in different lung regions in PET_MRLUNG. This table shows the SUVmean from ROI analysis in the different lung regions in the PET image reconstructed with the CT-based attenuation map in which the LACs of lung tissue were replaced by the respective LAC from the MR-based segmentation method (PET_MRLUNG).
(PDF)

S3 Table SUVmean in different lung regions in PET_MRLUNG_NOBONE. This table shows the SUVmean from ROI analysis in the different lung regions in the PET images reconstructed with the CT-based attenuation map in which the LACs of lung tissue and bone were replaced by the respective LACs from the MR-based segmentation method (PET_MRLUNG_NOBONE).
(PDF)

S4 Table SUVmean in different lung regions in PET_MRAC. This table shows the SUVmean from ROI analysis in the different lung regions in the PET image reconstructed with the MR attenuation map (PET_MRAC).
(PDF)

S5 Table Analysis of lung density. This table shows the results of the analysis of the mean lung density in different lung regions. This analysis takes a whole lung region into account. MLD = Mean lung density. SD = Standard deviation. AL/R = Apex left/right. ML/R = Middle left /right. BL/R = Basal left/right.
(PDF)

S6 Table Hounsfield units. This table shows the Hounsfield Units from ROI analysis in the CT images in the different lung regions, corresponding to the regions analysed in the PET images.
(PDF)
