# Supplementary material for: SUV-quantification of physiological lung tissue in an integrated PET/MR-system: Impact of lung density and bone tissue
Source: PLoS One. 2017 May 31;12(5):e0177856. doi: 10.1371/journal.pone.0177856 (PMC5451041; doi:10.1371/journal.pone.0177856)
Supplement: S1 Table — This table shows the SUVmean from ROI analysis in the different lung regions in the PET image reconstructed with the attenuation map from PET/CT (PET_CTAC). (PDF) [file pone.0177856.s001.pdf]

Table 1: SUVmean in different lung regions in PET_CTAC

Patients	1	2	3	4	5	6	7	8	9	10	11	12
**1**	0,38	0,45	0,62	0,39	0,34	0,58	0,36	0,37	0,44	0,32	0,40	0,57
**2**	0,32	0,40	1,02				0,32	0,42	0,69			
**3**	0,36	0,38	0,71	0,40	0,31	0,68	0,26	0,33	0,61	0,34	0,47	0,57
**4**	0,31	0,42	0,36	0,38	0,34	0,44	0,27	0,35	0,41	0,38	0,39	0,44
**5**	0,24	0,21	0,35	0,24	0,24	0,37	0,15	0,18	0,36	0,18	0,22	0,27
**6**	0,32	0,24	0,32	0,29	0,26	0,31	0,29	0,29	0,39	0,36	0,28	0,33
**7**	0,32	0,33	0,46	0,53	0,31	0,41	0,35	0,39	0,44	0,43	0,44	0,46
**8**	0,34	0,53	0,44	0,30	0,34	0,88						
**9**	0,19	0,19	0,29	0,22	0,22	0,33	0,21	0,22	0,26	0,24	0,24	0,28
**10**	0,32	0,29	0,56	0,40	0,48	0,59	0,30	0,32	0,41	0,44	0,42	0,66
**11**	0,28	0,28	0,27	0,25	0,24	0,23	0,23	0,23	0,23	0,23	0,23	0,24
**12**	0,43	0,31	0,49	0,27	0,38	0,73	0,34	0,39	0,39	0,28	0,53	0,53
**13**	0,34	0,34	0,61	0,34	0,38	0,56	0,32	0,34	0,91	0,24	0,34	0,65
**14**	0,37	0,32	0,44	0,41	0,37	0,44	0,26	0,31	0,37	0,39	0,47	0,67
**15**	0,22	0,29	0,36	0,26	0,25	0,34	0,22	0,16	0,29	0,27	0,25	0,47

No	Lung region
1-6	Hilus
7-12	Basal
1-3; 7-9	Right lung
4-6; 10-12	Left lung
1,4,7,10	anterior lung regions
2,5,8,11	middle lung regions
3,6,9,12	posterior lung regions
