# Supplementary material for: SUV-quantification of physiological lung tissue in an integrated PET/MR-system: Impact of lung density and bone tissue
Source: PLoS One. 2017 May 31;12(5):e0177856. doi: 10.1371/journal.pone.0177856 (PMC5451041; doi:10.1371/journal.pone.0177856)
Supplement: S2 Table — This table shows the SUVmean from ROI analysis in the different lung regions in the PET image reconstructed with the CT-based attenuation map in which the LACs of lung tissue were replaced by the respective LAC from the MR-based segmentation method (PET_MRLUNG). (PDF) [file pone.0177856.s002.pdf]

Table 2: SUVmean in different lung regions in PET_MRLUNG

Patients	1	2	3	4	5	6	7	8	9	10	11	12
1	0,38	0,47	0,62	0,39	0,35	0,57	0,41	0,38	0,43	0,33	0,41	0,55
2	0,35	0,39	0,70				0,40	0,36	0,51			
3	0,34	0,33	0,55	0,39	0,29	0,54	0,27	0,29	0,49	0,34	0,40	0,42
4	0,32	0,44	0,40	0,38	0,40	0,45	0,34	0,41	0,42	0,37	0,36	0,39
5	0,29	0,26	0,42	0,30	0,28	0,44	0,21	0,22	0,44	0,24	0,29	0,33
6	0,31	0,25	0,27	0,29	0,25	0,27	0,31	0,29	0,33	0,36	0,26	0,28
7	0,32	0,31	0,42	0,50	0,31	0,32	0,39	0,34	0,33	0,44	0,40	0,36
8	0,29	0,43	0,33	0,24	0,24	0,47						
9	0,19	0,20	0,26	0,26	0,21	0,33	0,24	0,21	0,23	0,25	0,25	0,26
10	0,34	0,31	0,55	0,44	0,44	0,55	0,32	0,34	0,43	0,45	0,40	0,49
11	0,27	0,27	0,26	0,24	0,23	0,23	0,22	0,22	0,22	0,22	0,22	0,24
12	0,43	0,32	0,43	0,34	0,43	0,66	0,34	0,43	0,35	0,32	0,43	0,37
13	0,33	0,32	0,55	0,34	0,37	0,51	0,33	0,33	0,78	0,26	0,34	0,54
14	0,33	0,34	0,38	0,36	0,33	0,40	0,28	0,28	0,30	0,36	0,44	0,45
15	0,28	0,34	0,37	0,31	0,29	0,36	0,28	0,21	0,32	0,33	0,27	0,50

No	Lung region
1-6	Hilus
7-12	Basal
1-3; 7-9	Right lung
4-6; 10-12	Left lung
1,4,7,10	anterior lung regions
2,5,8,11	middle lung regions
3,6,9,12	posterior lung regions
