# Supplementary material for: SUV-quantification of physiological lung tissue in an integrated PET/MR-system: Impact of lung density and bone tissue
Source: PLoS One. 2017 May 31;12(5):e0177856. doi: 10.1371/journal.pone.0177856 (PMC5451041; doi:10.1371/journal.pone.0177856)
Supplement: S3 Table — This table shows the SUVmean from ROI analysis in the different lung regions in the PET images reconstructed with the CT-based attenuation map in which the LACs of lung tissue and bone were replaced by the respective LACs from the MR-based segmentation method (PET_MRLUNG_NOBONE). (PDF) [file pone.0177856.s003.pdf]

Table 3: SUVmean in different lung regions in PET_MRLUNG_NOBONE

**Patients**	**1**	**2**	**3**	**4**	**5**	**6**	**7**	**8**	**9**	**10**	**11**	**12**
**1**	0,39	0,43	0,57	0,42	0,36	0,58	0,47	0,36	0,45	0,33	0,40	0,57
**2**	0,34	0,36	0,66				0,40	0,35	0,48			
**3**	0,39	0,32	0,50	0,40	0,30	0,48	0,32	0,31	0,44	0,39	0,39	0,38
**4**	0,32	0,45	0,39	0,40	0,40	0,45	0,35	0,37	0,38	0,37	0,39	0,38
**5**	0,28	0,23	0,36	0,30	0,27	0,42	0,20	0,20	0,40	0,25	0,30	0,32
**6**	0,29	0,27	0,29	0,27	0,26	0,28	0,27	0,30	0,34	0,35	0,27	0,28
**7**	0,35	0,32	0,41	0,50	0,34	0,32	0,38	0,29	0,30	0,47	0,43	0,33
**8**	0,33	0,42	0,31	0,22	0,21	0,41						
**9**	0,21	0,21	0,23	0,26	0,20	0,34	0,25	0,22	0,24	0,25	0,24	0,28
**10**	0,38	0,33	0,51	0,44	0,40	0,54	0,32	0,35	0,46	0,47	0,35	0,45
**11**	0,29	0,29	0,27	0,26	0,24	0,23	0,24	0,22	0,22	0,23	0,24	0,24
**12**	0,44	0,30	0,48	0,34	0,44	0,60	0,35	0,45	0,31	0,35	0,46	0,34
**13**	0,31	0,31	0,42	0,36	0,35	0,37	0,31	0,33	0,50	0,24	0,35	0,35
**14**	0,34	0,32	0,38	0,34	0,30	0,36	0,27	0,27	0,31	0,34	0,41	0,39
**15**	0,27	0,34	0,35	0,32	0,28	0,37	0,28	0,22	0,30	0,32	0,25	0,49

**No**	**Lung region**
1-6	Hilus
7-12	Basal
1-3; 7-9	Right lung
4-6; 10-12	Left lung
1,4,7,10	anterior lung regions
2,5,8,11	middle lung regions
3,6,9,12	posterior lung regions
