# Supplementary material for: SUV-quantification of physiological lung tissue in an integrated PET/MR-system: Impact of lung density and bone tissue
Source: PLoS One. 2017 May 31;12(5):e0177856. doi: 10.1371/journal.pone.0177856 (PMC5451041; doi:10.1371/journal.pone.0177856)
Supplement: S4 Table — This table shows the SUVmean from ROI analysis in the different lung regions in the PET image reconstructed with the MR attenuation map (PET_MRAC). (PDF) [file pone.0177856.s004.pdf]

Table 4: SUVmean in different lung regions in PET_MRAC

**Patients**	**1**	**2**	**3**	**4**	**5**	**6**	**7**	**8**	**9**	**10**	**11**	**12**
**1**	0,37	0,46	0,60	0,41	0,33	0,56	0,44	0,34	0,42	0,33	0,40	0,54
**2**	0,35	0,37	0,66				0,38	0,36	0,45			
**3**	0,36	0,33	0,54	0,41	0,28	0,51	0,30	0,29	0,47	0,36	0,38	0,38
**4**	0,33	0,41	0,38	0,39	0,37	0,42	0,34	0,39	0,39	0,38	0,37	0,36
**5**	0,28	0,26	0,38	0,29	0,26	0,41	0,20	0,21	0,40	0,23	0,27	0,31
**6**	0,30	0,25	0,26	0,26	0,25	0,27	0,29	0,29	0,32	0,35	0,26	0,27
**7**	0,34	0,31	0,38	0,46	0,32	0,33	0,38	0,29	0,29	0,46	0,40	0,32
**8**	0,31	0,41	0,30	0,22	0,22	0,45						
**9**	0,20	0,20	0,25	0,24	0,21	0,33	0,24	0,21	0,22	0,25	0,25	0,27
**10**	0,36	0,31	0,55	0,47	0,42	0,55	0,32	0,34	0,43	0,47	0,38	0,47
**11**	0,27	0,26	0,26	0,24	0,23	0,22	0,22	0,22	0,22	0,22	0,22	0,23
**12**	0,43	0,30	0,43	0,34	0,41	0,65	0,36	0,42	0,32	0,33	0,45	0,36
**13**	0,32	0,30	0,41	0,33	0,34	0,39	0,31	0,30	0,51	0,23	0,33	0,38
**14**	0,32	0,31	0,36	0,35	0,31	0,39	0,28	0,26	0,29	0,34	0,43	0,41
**15**	0,28	0,32	0,36	0,31	0,27	0,36	0,29	0,21	0,32	0,33	0,25	0,50

**No**	**Lung region**
1-6	Hilus
7-12	Basal
1-3; 7-9	Right lung
4-6; 10-12	Left lung
1,4,7,10	anterior lung regions
2,5,8,11	middle lung regions
3,6,9,12	posterior lung regions
