# Supplementary material for: SUV-quantification of physiological lung tissue in an integrated PET/MR-system: Impact of lung density and bone tissue
Source: PLoS One. 2017 May 31;12(5):e0177856. doi: 10.1371/journal.pone.0177856 (PMC5451041; doi:10.1371/journal.pone.0177856)
Supplement: S5 Table — This table shows the results of the analysis of the mean lung density in different lung regions. This analysis takes a whole lung region into account. MLD = Mean lung density. SD = Standard deviation. AL/R = Apex left/right. ML/R = Middle left /right. BL/R = Basal left/right. (PDF) [file pone.0177856.s005.pdf]

Table 5: Analysis of lung density

Patients	AL	ML	BL	AR	MR	BR	
1	-797	-811	-820	-819	-828	-828	MLD
	113	144	111	118	130	115	SD
2	-779	-721	-726	-805	-768	-766	MLD
	139	167	146	104	154	142	SD
3	-766	-770	-764	-779	-780	-765	MLD
	157	157	133	101	144	128	SD
4	-856	-830	-811	-856	-831	-835	MLD
	113	169	125	115	182	121	SD
5	-816	-846	-844	-820	-837	-856	MLD
	156	155	149	125	165	119	SD
6	-794	-784	-787	-788	-775	-782	MLD
	102	154	111	103	162	112	SD
7	-815	-799	-802	-817	-804	-809	MLD
	124	174	128	122	176	123	SD
8	-611	-572	-573	-657	-647	-624	MLD
	147	200	141	144	183	155	SD
9	-827	-836	-839	-820	-815	-844	MLD
	123	146	122	125	170	112	SD
10	-756	-729	-651	-771	-742	-718	MLD
	158	159	168	126	187	162	SD
11	-830	-817	-831	-827	-823	-834	MLD
	102	170	104	110	163	98	SD
12	-795	-788	-780	-786	-784	-777	MLD
	115	152	113	116	152	107	SD
13	-812	-788	-780	-806	-778	-762	MLD
	118	172	138	116	172	138	SD
14	-747	-728	-746	-760	-732	-757	MLD
	137	163	122	123	173	121	SD
15	-847	-824	-798	-857	-844	-845	MLD
	131	178	167	116	169	128	SD
