# Supplementary material for: SUV-quantification of physiological lung tissue in an integrated PET/MR-system: Impact of lung density and bone tissue
Source: PLoS One. 2017 May 31;12(5):e0177856. doi: 10.1371/journal.pone.0177856 (PMC5451041; doi:10.1371/journal.pone.0177856)
Supplement: S6 Table — This table shows the Hounsfield Units from ROI analysis in the CT images in the different lung regions, corresponding to the regions analysed in the PET images. (PDF) [file pone.0177856.s006.pdf]

Table 6: Hounsfield Units

Patients	1	2	3	4	5	6	7	8	9	10	11	12
1	-747,6	-800,4	-773,9	-780,0	-768,8	-774,6	-814,4	-674,8	-757,8	-769,7	-704,3	-757,6
2	-823,7	-760,5	-545,0				-842,6	-575,7	-661,6			
3	-733,0	-740,2	-668,8	-720,5	-747,7	-656,2	-804,7	-710,4	-684,1	-708,0	-703,8	-562,9
4	-793,1	-798,9	-775,2	-797,0	-777,0	-801,0	-814,1	-833,8	-716,2	-863,0	-802,7	-786,0
5	-846,7	-866,2	-829,1	-849,8	-785,4	-854,1	-895,1	-715,4	-857,6	-881,7	-891,8	-844,4
6	-767,9	-796,8	-666,1	-763,1	-769,3	-739,9	-816,7	-770,5	-675,4	-779,7	-749,5	-664,7
7	-776,2	-666,2	-691,3	-755,0	-818,7	-668,0	-766,5	-632,6	-568,6	-782,4	-736,0	-732,3
8	-715,9	-741,2	-611,6	-716,5	-633,3	-389,3						
9	-802,4	-787,7	-648,7	-810,8	-778,5	-796,2	-827,1	-725,3	-730,7	-763,2	-800,6	-745,2
10	-770,3	-754,3	-709,5	-818,9	-776,1	-763,2	-775,8	-794,9	-794,9	-783,2	-770,3	-552,0
11	-778,2	-753,4	-595,5	-775,1	-715,8	-518,8	-825,2	-727,2	-825,2	-755,3	-678,9	-509,9
12	-708,5	-749,4	-709,6	-848,6	-753,9	-732,2	-747,4	-794,2	-734,7	-749,1	-596,8	-716,2
13	-774,3	-693,7	-502,8	-777,6	-698,5	-621,6	-764,7	-751,2	-393,2	-769,1	-760,1	-522,2
14	-682,0	-807,8	-719,5	-665,0	-741,1	-747,4	-777,3	-759,0	-637,5	-715,0	-725,0	-575,6
15	-850,9	-855,6	-800,2	-858,6	-766,9	-855,0	-879,7	-883,6	-696,9	-832,0	-769,6	-842,3

**No**	**Lung region**
1-6	Hilus
7-12	Basal
1-3; 7-9	Right lung
4-6; 10-12	Left lung
1,4,7,10	anterior lung regions
2,5,8,11	middle lung regions
3,6,9,12	posterior lung regions

Patients examined with contrast-enhanced CT

Patients examined with non-contrast-enhanced CT
